# Supplementary material for: Identification of photoperiod-induced specific miRNAs in the adrenal glands of Sunite sheep (Ovis aries)
Source: Front Vet Sci. 2022 Jul 22;9:888207. doi: 10.3389/fvets.2022.888207 (PMC9354845; doi:10.3389/fvets.2022.888207)
Supplement: Supplementary file 1 [file Data_Sheet_1.ZIP › Supplementary file/miRDeep2 file.zip!/miRDeep2 file/Novel_Structure/SPLP42A1/Novel_617.pdf]

Provisional ID	:	NC_019471.2_20119
Score total	:	7.2
Score for star read(s)	:	3.9
Score for read counts	:	-0.3
Score for mfe	:	2.1
Score for randfold	:	1.6
Score for cons. seed	:	
Total read count	:	11
Mature read count	:	10
Loop read count	:	0
Star read count	:	1

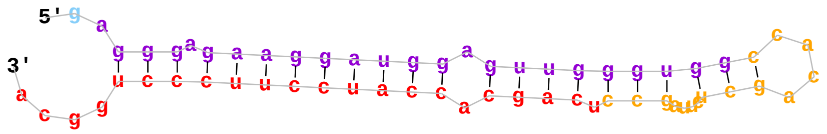

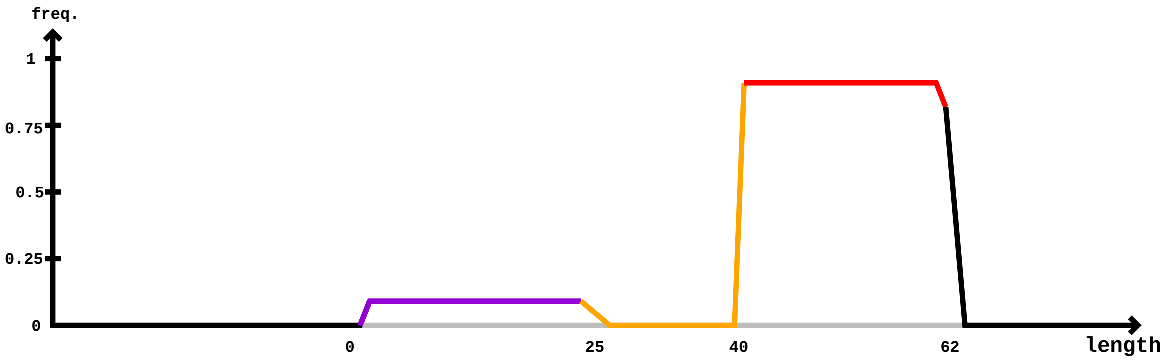

Star Mature

```
5'- gcuggguccugagccacuggggucagcagugagggagaaggauggaguugggugguccacagcucuuagccucagcaccauccuucccuggcaccacugccccuucuugagc   -3'    obs
    gcuggguccugagccacuggggucagcagugagggagaaggauggaguugggugggccacagcucuuagccucagcaccauccuucccuggcaccacugccccuucuugagc          exp
    (((........)))....((((.(((..(((.(((.(((((((((.((((((((((....)))....))).)))).))))))))))))..)))..)))))))).........   reads   mm   sample
    ...............................agggagaaggauggaguuggguggg........................................................   1       0    seq
    ......................................................................ucagcaccauccuucccuggc....................   1       0    seq
    ......................................................................ucagcaccauccuucccuggca...................   8       0    seq
    ......................................................................ucagcaccauccuucccuggcac..................   1       0    seq
```
